# Where Do Phosphosites Come from and Where Do They Go after Gene Duplication?

**DOI:** 10.1155/2012/843167

**Published:** 2012-06-20

**Authors:** Guillaume Diss, Luca Freschi, Christian R Landry

**Affiliations:** Département de Biologie, PROTEO and Institut de Biologie Intégrative et des Systèmes, Université Laval, Pavillon Charles-Eugène-Marchand, 1030, Avenue de la Médecine, Québec, QC, Canada G1V 0A6

## Abstract

Gene duplication followed by divergence is an important mechanism that leads to molecular innovation. Divergence of paralogous genes can be achieved at functional and regulatory levels. Whereas regulatory divergence at the transcriptional level is well documented, little is known about divergence of posttranslational modifications (PTMs). Protein phosphorylation, one of the most important PTMs, has recently been shown to be an important determinant of the retention of paralogous genes. Here we test whether gains and losses of phosphorylated amino acids after gene duplication may specifically modify the regulation of these duplicated proteins. We show that when phosphosites are lost in one paralog, transitions from phosphorylated serines and threonines are significantly biased toward negatively charged amino acids, which can mimic their phosphorylated status in a constitutive manner. Our analyses support the hypothesis that divergence between paralogs can be generated by a loss of the posttranslational regulatory control on a function rather than by the complete loss of the function itself. Surprisingly, these favoured transitions cannot be reached by single mutational steps, which suggests that the function of a phosphosite needs to be completely abolished before it is restored through substitution by these phosphomimetic residues. We conclude by discussing how gene duplication could facilitate the transitions between phosphorylated and phosphomimetic amino acids.

## 1. Introduction

Gene duplication is one of the most prominent mechanisms by which organisms acquire new functions [1]. Spectacular examples of such gains of function resulting from gene duplications are the evolution of trichromatic vision in primates [2], the evolution of human beta-globin genes that are involved in the oxygen transport at different developmental stages [3] as well as the expansion of the family of immunoglobulins and other immunity-related genes that shaped the vertebrate immune system [4, 5]. Because of the central role of gene duplication in evolution, there has been a profound interest for a better understanding of how these new functions evolve at the molecular level [6], for determining at what rate gene duplication occurs [7–9] and for testing whether the retention of paralogous genes necessarily requires the evolution of new functions [6, 10, 11]. One of the most important challenges has been to determine mechanistically how specific mutations translate into new functions, as establishing sequence-function relationships remains a difficult task [12].

After a gene duplication event, the two sister paralogs are identical copies of their ancestor and encode two identical functions, thus relaxing the selective constraints on each paralog [8]. Under most evolutionary models, both paralogs have to diverge to be retained on evolutionary time scales, otherwise one paralog would be lost and the system would return to its ancestral state (nonfunctionalization) [6]. There are two ways for paralogs to diverge in function. The first one is the acquisition of new functions by one or both of the two paralogs, a mechanism called neofunctionalization [1, 8, 10]. The second mechanism, called subfunctionalization, implies the complementary partitioning of the ancestral function between the two paralogs by losses of functions [8, 10, 13]. These two mechanisms are not mutually exclusive because the ancestral function can be partitioned by subfunctionalization and then one or both paralogs may acquire new functions by neofunctionalization, a mechanism called neosubfunctionalization [14]. An increase in the dosage of a gene product by the addition of a second identical copy of the ancestral gene can also contribute to the retention of paralogous pairs, without the need for the gain or loss of functions [15, 16]. 

Divergence between paralogs does not necessarily imply a divergence in a specific function but can also involve a change in the regulation of that function. For instance, the regulatory control of a protein function can be modified at the transcriptional or at the posttranslational level. Divergence in expression pattern of duplicated transcript is well documented [1, 10, 17, 18]. For example, Gu et al. showed that a large fraction of ancient duplicated gene pairs in yeast shows divergent gene expression patterns [18]. A more recent study showed that nearly half of the genes that duplicated after a whole genome duplication event (WGD) in a forest tree species have diverged in expression by a random degeneration process [19]. However, little is known about the divergence of regulation by posttranslational modifications (PTMs), which take place after transcription and translation and directly affect protein activities [20].

PTMs are covalent modifications of one or more amino acids that affect the activity of a protein, its localization in the cell, its turnover rate, and its interactions with other molecules [21]. Cells use a wide range of different PTMs to exert distinct regulations on proteins. Although only 20 amino acids are encoded by the genetic code, more than 200 amino acid variants or their derivatives are found in proteins after PTMs [22]. Phosphorylation, the addition of a phosphate moiety from an ATP donor to a serine (Ser), threonine (Thr), or tyrosine (Tyr) residue by a protein kinase, is by far the best-known PTM, as it is the most common and is involved in the regulation of key biological processes of fundamental and medical interest, such as signal transduction and cell-cycle regulation [23]. Phosphorylation of these amino acids modifies their biochemical properties in several manners. Of particular interest for this study is the addition of a phosphate group that brings two new negative charges that allow the formation of a salt bridge or that contribute to the local charge of the protein [24]. Given that a phosphate group is a relatively large molecule, phosphorylation can also have sterical effects. Such properties can notably induce conformational changes of the protein, modify its catalytic activity, or block the access to its catalytic site, which result in the activation or inhibition of the activity of the target protein by direct or allosteric effects [24].

Several of the effects of protein phosphorylation can be mimicked by the negatively charged amino acids aspartic acid (Asp) and glutamic acid (Glu). Indeed, the biochemical properties of these amino acids are close to those of phosphorylated Ser or Thr residues [25]. In particular conditions, Asp and Glu are constitutive functional equivalents of phosphosites in a phosphorylated state. This functional resemblance has been exploited by biochemists by replacing Ser and Thr residues by Asp and Glu in proteins of interest in order to mimic their phosphorylated status. This molecular mimicry led them to call Asp and Glu phosphomimetic amino acids [25]. This trick appears to have been also used by nature to evolve new phosphosites. A striking example comes from the evolution of the Activation Induced cytidine Deaminase (AID) across vertebrates, an enzyme involved in the generation of antibody diversity. The interaction of this enzyme with the Replication Protein A (RPA) promotes AID access to transcribed double-stranded DNA during immunoglobulin class switch recombination. This interaction requires a negative charge on AID, which is provided by an Asp in bony fish. In these organisms, the enzyme is constitutively capable of interacting with RPA. In amphibians and mammals, the function of the Asp residue is carried out by a phosphorylatable Ser (pSer), which allows the regulation of the protein interaction by protein kinases in a condition-specific fashion [26]. It was recently suggested that this type of evolutionary transitions might be common. Globally, it was shown that pSer tends to evolve from or to phosphomimetic amino acids (Asp and Glu) when gained and lost, respectively, throughout the evolution of eukaryotes [27, 28].

Protein phosphoregulation has been suggested to play a role in the evolutionary fate of paralogous proteins. Most studies done so far focused on the paralogous genes of the budding yeast *Saccharomyces cerevisiae* because its phosphoproteome has been intensely studied [29–31]. Using the yeast paralogs that derive from the WGD event, Amoutzias et al. showed that the number of phosphosites on a phosphoprotein is an important determinant for the retention of its duplicated descendants [32]. In a following study, Freschi et al. studied the gains and losses of phosphosites in paralogous phosphoproteins and found that the great majority of them are present in one paralog and not in the other. This divergence was shown to be principally driven by losses rather than gains of phosphosites on one paralog [33]. Finally, Kaganovich and Snyder found that phosphosites tend to diverge more asymmetrically than nonphosphorylated amino acids, playing thus an important role in paralogous genes divergence and retention [34]. These observations raise the question of where do phosphosites come from and where do they go after a gene duplication. According to the observations on phosphomimetic amino acids described above, gains and losses of phosphosites could represent two distinct types of divergence. On the one hand, the gain or the loss of phosphosites from or to a nonphosphomimetic residue would represent a divergence in the function of the protein. On the other hand, a gain or a loss could occur from or to phosphomimetic residues, leading to a modification of the control of the charged residue by the cell rather than a modification of function per se. Here we test whether this second scenario could have contributed to the divergence of paralogous proteins using the yeast phosphoproteome as a model. 

## 2. Methods

### 2.1. Dataset

All analyses were performed using the dataset we compiled in a previous study [33], and that is available at http://www.bio.ulaval.ca/landrylab/download/ (Dataset 1). This dataset contains 20,342 phosphosites on 2688 proteins from eight large-scale studies [29–31, 35–39]. It also provides the alignments of all *S*. *cerevisiae* WGD paralogous genes with their ancestral sequence and with the orthologs of *Lachancea kluyveri* and *Zygosaccharomyces rouxii. *The alignments were performed using MUSCLE [40] while the ancestral sequence was inferred using the Codeml method implemented in PAML [41]. We chose to analyze only two species that diverged before the WGD event for the following reasons. The majority of phosphorylation sites are located in disordered regions [42], and these regions are fast evolving. Alignment of sequences from distantly related species leads to spurious alignments or to alignments that may contain several indels. Indels decrease the number of phosphorylation sites available for the analysis, as ancestral sequences cannot be computed at these positions. Further, in Freschi et al. [33], we performed the analyses including an additional species that diverged prior to the whole-genome duplication, and we found that this did not significantly affect our results. Finally, this dataset also provides information about the localization of each residue in ordered or disordered regions of the protein, according to predictions made with DISOPRED [43]. 

### 2.2. Approaches to Study Gains and Losses of Phosphosites

**Figure 1:**
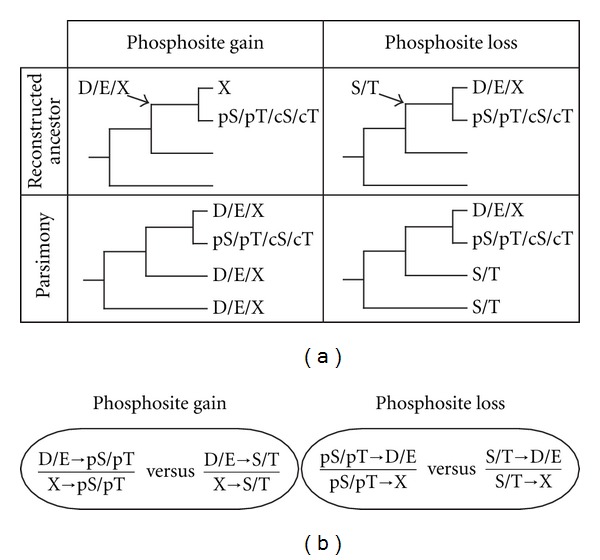
Algorithm used to calculate and compare the proportions of transitions between phosphorylated and phosphomimetic residues relative to control sites. (a) Phosphosite (pS, pT) gains from phosphomimetic amino acids were identified as cases where only one of the paralog has a phosphosite and the ancestral sequence has a phosphomimetic residue at the same position. Control sites (cS, cT) were identified in the same way but considering Ser and Thr that are not known to be phosphorylated. The ancestral sequence was inferred using likelihood or parsimony approaches. Phosphosites losses to phosphomimetic amino acids were identified as cases where one paralog has a phosphosite in a position that is occupied by a phosphomimetic amino acid in the other paralog and a phosphorylatable amino acid at the same position in the ancestral sequence. (b) The proportion of pS or pT that evolved from or to D or E was compared to the proportion of cS or cT that evolved from or to D or E. X represents any amino acid with the exception of Ser, Thr and Tyr.

We applied different approaches to study gains and losses coming from or going to negatively charged amino acids. In the first approach, we used the ancestral sequence as a reference to assess the presence of a gain or a loss at a specific position. For the gains, we compared the proportion of phosphomimetic amino acids in the ancestral sequence (Asp or Glu) going to pSer or pThr to the proportion of phosphomimetic amino acids going to cSer and cThr. For the losses, we compared the proportion of phosphorylated residues (pSer and pThr) coming to Asp or Glu to the proportion of nonphosphorylated residues (cSer and cThr) coming to Asp or Glu, respectively. We required the ancestral sequence to have a phosphorylatable residue and one of the two paralogs to be phosphorylated at the homologous position. Comparisons of proportions were performed using Fisher's exact tests as implemented in R [44]. In our second approach, we used a parsimony method to calculate the same proportions. This time we used the sequences of *L. kluyveri* and *Z. rouxii* as reference. In the case of a gain of phosphosites, we required the presence of the same negatively charged residue (Asp or Glu) in the reference species as well as in one of the two paralogs and a phosphorylatable residue (Ser or Thr) in the other paralog. In the case of losses of phosphosites, we required the presence of the same phosphorylatable residue (Ser or Thr) in the reference species as well as in one of the two paralogs and a negatively charged residue (Asp or Glu) in the other paralog. All proportions were calculated by dividing the number of sites coming from or going to an Asp or a Glu by the number of sites that come from or go to any of the 17 nonphosphorylatable amino acids following the same criteria (Figure 1).

## 3. Results

The phosphoproteome of *S. cerevisiae* is the best described among eukaryotes and has been mapped by mass spectrometry, leading to the identification of high-confidence phosphosites [29–31]. We assembled a data set [33] that consists of 2,726 phosphosites (Ser, 82%; Thr, 16%; Tyr, 2%) that belong to one or the other member of the 352 pairs of yeast WGD paralogs for which at least one of the two proteins is a phosphoprotein. We inferred the ancestral sequence for each pair of paralogs using alignments with orthologous sequences from *L. kluyveri* and *Z. rouxii*, two species that diverged from *S. cerevisiae* before the WGD event. For each pair, we aligned all five sequences, we mapped the phosphosites on the sequences of the paralogs and analysed phosphosites that diverged, that is, cases where a phosphorylatable residue was present in only one paralog.

**Figure 2:**
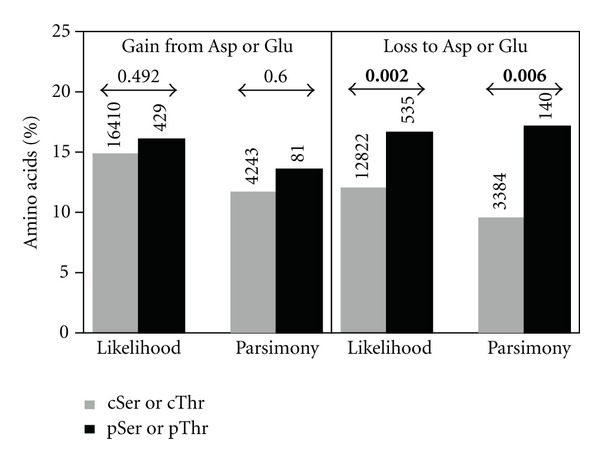
Phosphosites that are differentially lost in paralogous phosphoproteins evolve toward negatively charged residues. Each bar represents the percentage of sites (pSer and pThr, cSer and cThr) that evolved from or to Asp or Glu. Numbers above the bars represent the total number of pSer, cSer, pThr, or cThr sites that were gained or lost. Numbers above the arrows indicate *P*-values of the Fisher's exact tests, bold ones being below 0.05.

**Figure 3:**
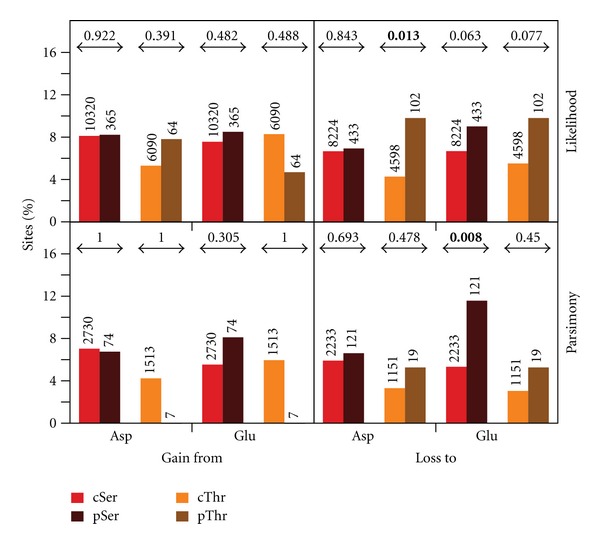
Detailed analysis of the patterns of evolution of pSer and pThr sites. Each bar represents the percentage of sites (pSer, cSer, pThr, or cThr) that evolved from or to Asp or Glu. Numbers above the bars represent the total number of pSer, cSer, pThr, or cThr sites that were gained or lost. Numbers above the arrows indicate *P*-values of the Fisher's exact tests, bold ones being below 0.05. The top panel shows results obtained by ancestral sequence reconstruction using a likelihood approach and the bottom panel using parsimony.

Under a scenario where gains of phosphosites would result from selection for transitions from phosphomimetic amino acids to phosphorylated residues, we would expect phosphorylated Ser or Thr (pSer and pThr, resp.) to evolve more often from Asp or Glu than nonphosphorylated ones (cSer and cThr, resp.). Similarly, under a scenario where losses of phosphosites would result from transitions from phosphorylated residues to phosphomimetic amino acids, we would expect pSer and pThr to evolve more often to Asp and Glu than equivalent cSer and cThr. We tested these two hypotheses as described in Figure 1. In the first case, we compared the proportion of pSer and pThr that were gained from Asp and Glu with that of cSer and cThr, that is, all serines and threonines from the same set of proteins that were gained from Asp and Glu but that are not known to be phosphorylated. In the second case, we compared the ratio of sites that were lost and replaced by phosphomimetic residues in only one paralog with the ratios derived from cSer and cThr. We performed the analysis using paralogous ancestral sequences inferred with a likelihood method and also using a parsimonious approach, whereby the ancestral state of phosphosites was inferred based on the conservation of the site in one of the two paralogs and its two orthologs (Figure 1(a)). Global results are presented in Figure 2, and detailed analyses are presented in Figure 3. 

A gobal analysis of pSer, pThr, Asp, and Glu shows that phosphosites tend to be lost to Asp and Glu more frequently than cSer and cThr, and this holds true for both likelihood (16.6% versus 12.1%, resp., *P* = 0.002) and parsimony (17.1% versus 9.6%, resp., *P* = 0.006) reconstruction methods (Figure 2). However, although there is a tendency towards the gains of phosphosites from Asp and Glu, the observed differences are not significant (Figure 2). When studied separately, phosphosites in ordered and disordered regions show the same global tendency to go toward phosphomimetic amino acids (likelihood: 17.5% versus 10.0% in ordered regions, *P* = 0.058; 16.5% versus 13.7% in disordered regions, *P* = 0.086, parsimony: 20.0% versus 8.1% in ordered regions, *P* = 0.076; 16.7% versus 11.7% in disordered regions, *P* = 0.110). Further, we found that phosphosites are not preferentially gained from phosphomimetic amino acids in disordered regions, while there is a nonsignificant tendency for this type of transition in ordered regions (likelihood: 16.0% versus 15.7% in disordered regions, *P* = 0.943; 18.8% versus 13.7% in ordered regions, *P* = 0.294, parsimony: 14.1% versus 14.2% in disordered regions, *P* = 1.000; 11.8% versus 10.2% in ordered regions, *P* = 0.691). This suggests that the effect might be more important in ordered regions of proteins, as would be expected if these residues were playing structural roles. Because the distinction between order and disorder reduces the number sites in each category and does not provide opposite results, we considered both regions simultaneously in the following analyses.

We also examined which class of substitution could be contributing to this overall result (Figure 3). We first found that pSer and pThr that were gained after gene duplication follow trends that are in the expected direction although some of the comparisons are not statistically significant and other results are in the opposite direction (Figure 3). However, this detailed analysis showed that pSer is significantly more likely to evolve to Glu than cSer (11.6% versus 5.3%, *P* = 0.008) while pThr evolves significantly more frequently to Asp than cThr (9.8% versus 4.3% resp., *P* = 0.013). 

## 4. Discussion

Protein phosphorylation is known to have a key role in regulating protein activities [45]. Evolutionary events such as gains and losses of phosphosites can lead to changes in protein regulation, thus rewiring the protein regulatory network of the cell [33]. In the literature, there is evidence for gains of new phosphosites coming from negatively charged residues among orthologs [26, 27] as well as cases of losses of phosphosites to these amino acids [28]. The biochemical properties of Glu and Asp mimic the ones of pSer and pThr with the exception that their charge is not regulatable [25]. These observations led us to hypothesize that coding sequence divergence of paralogous genes by neo- and subfunctionalization does not strictly involve the apparition or the partitioning of protein function. Paralogous genes could also diverge in how these functions are regulated. Divergence in the regulatory control is well known at the transcriptional level [19, 46] but has not been specifically addressed at the posttranslational level. We tested this hypothesis on the complete set of WGD phosphoproteins of the budding yeast *S. cerevisiae*.

Using two different methods to infer the ancestral state of phosphorylated and nonphosphorylated Ser and Thr, we found that pSer and pThr globally have a tendency to evolve from negatively charged amino acids in paralogous phosphoproteins compared to their nonphosphorylated counterparts. The tendencies observed are in agreement with our hypothesis and with the observations made by Pearlman et al. across eukaryotes [27]. However, the observed differences are not significant, which could be explained by a few nonexclusive scenarios. First, we are looking at a narrow evolutionary window (100 My), which contrasts with the analysis conducted by Pearlman et al., who used aligned sequences from organisms spanning the entire tree of life [27]. Further, the mechanism proposed may apply primarily to few sites and in ordered regions of proteins. Only few phosphosites in these regions could be analysed here since the majority of them are found in disordered regions [42], which reduces the statistical power of our analysis. Our results regarding gains of phosphosites are in line with this hypothesis. Finally, a significant fraction of phosphosites are thought to be nonfunctional [42]. Because these nonfunctional sites are not under selective pressure, they may contribute to decrease the signal coming from functional sites. Nevertheless, from our results, we cannot rule out the possibility that gains of phosphosites are not more likely to derive from phosphomimetic residues after gene duplications. A larger sample size, the study of a time window of a different length and a better knowledge of the functional importance of phosphosites may be needed to provide a final answer.

Following the same approach, we examined whether phosphorylated residues, when lost, are more likely to be replaced by Asp and Glu than when nonphosphorylated equivalent residues are lost. We found that this is the case globally and also when considering individual cases for both pSer and pThr; pSer are more likely to be replaced by Glu residues while pThr by Asp residues. A similar trend was detectable for the transitions from pThr to Glu. These results are in agreement with those from Kurmangaliyev et al. [28] who also showed that pSer are more likely to evolve to phosphomimetic amino acids than cSer in the divergence of orthologs between species. Our results show that the evolutionary trajectories of pSer and pThr provide a mechanism for paralogous protein divergence. Our analyses support the hypothesis that divergence between paralogs can be generated by a loss of the posttranslational regulatory control on a function rather than by the complete loss of the function itself. Indeed, the substitution of a phosphosite for an Asp or a Glu residue may block one paralog into a single constitutive functional state whereas the other one remains regulatable by protein kinases and phosphatases. 

**Figure 4:**
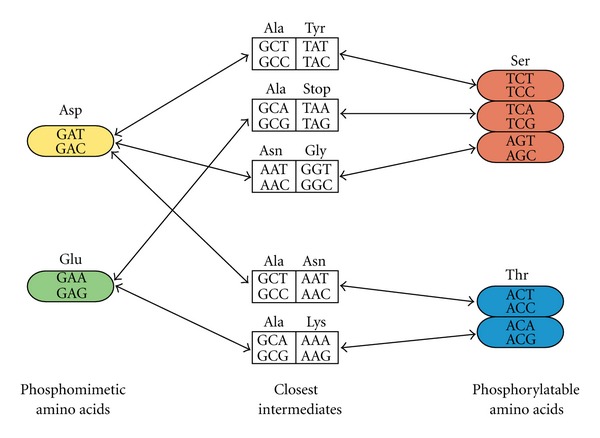
Transitions between phosphorylatable and phosphomimetic amino acids need to go through a nonnegatively charged intermediate.

**Figure 5:**
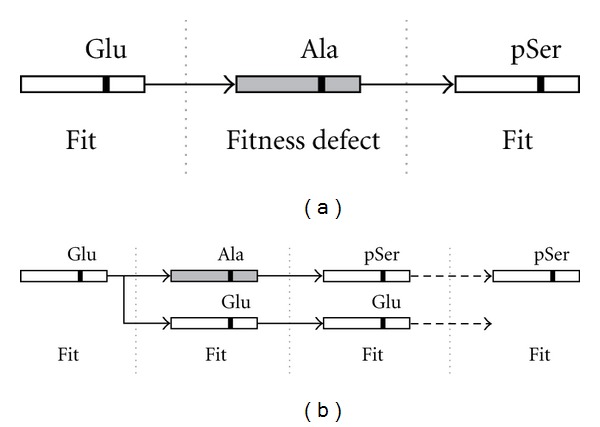
A duplication event could provide the conditions for the intermediate nonfunctional site to be neutral, which would allow a transition without affecting the fitness of the organism. (a) Without a duplication event, the loss of a negative charge could have deleterious effects if the charge is important for the function of the protein. (b) The redundant paralogous gene copy could serve as a backup and prevent deleterious effects created by the loss of the charge. The backup copy could then be retained or lost. In the latter case, the system would be different from its ancestor.

Our results raise the question of how these transitions are made possible during evolution. The genetic code is organized in such a way that transitions between phosphorylatable and phosphomimetic amino acids involve a transition state with an amino acid that is not negatively charged, except for transitions between two Asp and two Ser codons that involve a Tyr residue (Figure 4). However, Tyr is only rarely phosphorylated in yeast, and Tyr residues are not phosphorylated by the serine/threonine kinases [47], which suggests that this path would not be favoured. Our results also suggest that this evolutionary route is uncommon. A non negatively charged intermediate could lead to a complete loss of the function that was performed by the negative charge and could thus be deleterious (Figure 5(a)). Here we propose that the relaxed constraints that follow a gene duplication event could provide the mean to reach this intermediate state and to go beyond (Figure 5(b)). After gene duplication, when one of the duplicated copies is lost, the system is assumed to go back to its ancestral state, a process called nonfunctionalization [8]. However, following our model, the duplicated copy could serve as a backup for a transition period, which would allow the other copy to reach a state that would have been unreachable otherwise [48–50]. After the loss of the backup copy, the system would remain different from its ancestral state since the phosphorylation profile and thus the phosphoregulation of this protein has changed. The term nonfunctionalization may thus not be suitable for such cases. In the case of a WGD event, where the vast majority of the duplicated genes are eventually lost and are thought to return back to their ancestral state, these 2-step transitions could potentially lead to a great burst in the evolution of phosphoregulation. Further studies at different time points following gene duplication would be needed to determine how important this mechanism could be for the evolution of phosphorylation networks.
